# Physical risk factors associated with central serous chorioretinopathy: A protocol for a systematic review and meta-analysis

**DOI:** 10.1371/journal.pone.0349473

**Published:** 2026-10-29

**Authors:** Olivia Cundy, William Wilson, Catey Bunce, Timothy L. Jackson

**Affiliations:** 1 Faculty of Life Sciences and Medicine, King’s College London, London, United Kingdom; 2 King’s Ophthalmology Research Unit, King’s College Hospital, London, United Kingdom; 3 Cancer Research UK & UCL Cancer Trials Centre, London, United Kingdom; 4 London School of Hygiene and Tropical Medicine, London, United Kingdom; Icahn School of Medicine at Mount Sinai, UNITED STATES OF AMERICA

## Abstract

**Background:**

Central serous chorioretinopathy (CSCR) is a common vision-threatening retinal disease that affects predominantly working-age adults. Although often self-limiting, a substantial proportion of cases are either chronic or recurrent, resulting in significant visual, psychological, social and economic burdens on sufferers. Current treatment options are limited and lack robust long-term evidence, reflecting an incomplete understanding of underlying disease mechanisms. Traditionally, CSCR has been researched as a solely ocular condition, however numerous non-ophthalmic physical risk factors have been implicated in the disease, suggesting a broader systemic aetiology. A comprehensive synthesis of physical risk factors for CSCR is needed to inform future mechanistic research and therapeutic development.

**Objective:**

The main objective of this systematic review and meta-analysis is to summarise physical risk factors associated with CSCR, including genetic, hormonal, metabolic and drug- or toxin-related factors. The secondary objective is to describe any associations between individual risk factors and disease severity.

**Methods and analysis:**

This protocol has been written in accordance with the Preferred Reporting Items for Systematic review and Meta-Analysis Protocols (PRSIMA-P). The PRISMA-P Checklist for this protocol can be found in Supplemental Information S2: PRISMA-P Checklist. Observational and interventional studies that report on any physical risk factors for CSCR will be included. Eligible participants are adults (≥ 18 years) with CSCR. Searches will be conducted on MEDLINE, Embase, CINAHL, Web of Science, Scopus and CENTRAL, and supplemented by grey literature and clinical trial registry searches. Two reviewers will independently screen studies, extract data and assess the risk of bias, with any disagreements adjudicated by a third reviewer. Where feasible, meta-analyses will be performed using random-effects models to pool odds ratios for associations between risk factors and CSCR. Heterogeneity of the included studies will be assessed using the I^2^ statistic. A narrative synthesis will be undertaken when meta-analysis is not possible, in accordance with Synthesis Without Meta-Analysis (SWiM) guidelines. Risk of bias will be assessed using the Newcastle-Ottawa Scale. The certainty of evidence will be evaluated using the Grading of Recommendations, Assessment, Development, and Evaluation (GRADE) system.

**Ethics and dissemination:**

Ethical approval is not required as this study uses previously published aggregate data. Findings will be disseminated through publication in peer-reviewed journal and presentation at relevant scientific conferences.

**Trial registration:**

This review protocol is registered with the International Prospective Register of Systematic Reviews (PROSPERO), under registration number CRD420251088489.

## Introduction

### Rationale

Central serous chorioretinopathy (CSCR) is a disease in which fluid accumulates underneath the neuroretina, the light-sensing layer of cells inside the back of the eye. This fluid causes blurred central vision, which can greatly impact reading, work, and driving. In up to 40% of people it affects both eyes [1,2] Central serous chorioretinopathy is the fourth most common vision-threatening retinal disorder in the UK and usually occurs in those of working age, from 30–50 years old [3]. Typically, CSCR resolves over the course of 6–12 months, however 1 in 6 cases lasts longer, and up to half recur [4–7] Because of the central location of visual loss caused by CSCR and the age at which it commonly presents (during working years), it can have a particularly significant psychological, social and economic impact on sufferers.

There is currently an insufficient understanding of the mechanisms underlying CSCR, and this may underlie a consequent paucity of effective, evidence-based treatments. None of the currently available treatments for CSCR are licensed. Laser- and light-based treatments are used to treat CSCR, but have yet to be proven effective in long-term high-quality trials, and carry the risk of sight loss [8,9] The largest, high-quality drug trial failed to show efficacy [10]. We need a better understanding of the underlying cause of CSCR, if we are to develop an effective treatment.

Most research on CSCR has focused on the eye, however there are compelling reasons to consider wider underlying causes. For example, many of the already identified risk factors for CSCR are not ophthalmic, including male sex, high levels of steroids in the blood stream, stress, driven personalities (historically termed “Type A”) and sleep disturbance [2,11–19]. Hence, CSCR may not be an “eye” disease *per se*, but a whole-person disorder manifesting in the eye. Consequently, in addition to drugs and device therapies, psychological therapies and lifestyle changes may have therapeutic potential.

We propose an in-depth review of the literature to summarise what is currently known about the systemic physical (i.e., non-psychological, biological) risk factors associated with CSCR. A separate review by the same research team will address psychological risk factors.

A greater understanding of the pathogenesis of CSCR may guide strategies designed to develop new and more effective treatments.

### Objectives

The main objective of this systematic review and meta-analysis is to summarise what physical risk factors are associated with CSCR, including both previously established factors and less well-recognised, potentially novel factors. The secondary objective is to describe any relationship(s) between individual risk factors and the degree of disease severity.

To this end, the proposed systematic review will answer the following questions:

1. What genetic, hormonal, metabolic, drug/toxin-related, and other risk factors are associated with CSCR in adult humans?
2. Is there a correlation between any individual risk factor for CSCR and ophthalmic features of disease severity?

## Methods

### Eligibility criteria

Studies will be selected according to the criteria outlined below.

### Study designs

We will include the following types of observational study: case-control, cohort, cross-sectional, case series and case reports. Interventional studies will only be included if they also report on risk factors associated with CSCR, e.g., in a sub-analysis. Interventional study types that will be included are randomised controlled trials (RCTs), non-randomised controlled clinical trials, pilot trials and feasibility trials. Studies with and without control groups will be included, although only the results from those with control groups deemed at low risk of bias will be used in the meta-analysis. We will also include letters and opinion pieces, as these might report on observed associations between biological factors and disease, although not in the formal framework of any of the above study types.

### Participants

We will include studies examining human adults (> 18 years old) diagnosed with active fovea-involving CSCR (based on history, examination and OCT scan, + /- fundus fluorescein angiography [FFA]).

### Outcomes of interest

We do not have a specific list of systemic biological associations for inclusion / exclusion, as one aim of this review is to identify new associations (in addition to those that have already been described). We categorize factors into genetic, hormonal, metabolic and drug or toxins, and provide some examples of each category. Genetic factors might include specific gene mutations or whole genome descriptions. Hormonal factors might be, for example, serum or urinary cortisol levels, or serum testosterone levels. Metabolic factors could include markers of lipid metabolism, for example levels of serum fatty acid amines. Drugs- or toxin-related factors could include exogenous anabolic androgenic steroids (AAS). We will also include studies that report on other physical factors that do not directly fall into one of the above categories, for example medical comorbidities such as hypertension or sleep apnoea, or demographic features for example latitude or ethnicity. These will be grouped into a separate category ‘Other’.

### Publication date

We will include studies published from 1991 (the year of the introduction of OCT scanning) in order to ensure included participants meet all eligibility criteria.

### Publication type

We will exclude editorials, non-peer reviewed articles, abstract-only articles, laboratory-only and non-human studies.

### Setting

There will be no restrictions on type of setting.

### Language

We will include studies originally published in other languages other than English, if it is possible to translate them into English. Non-English language studies will be translated into English using an online translation engine at the title and abstract screening stage. Those that are deemed potentially eligible for inclusion in the review will then have their full text translated to English prior to screening for inclusion.

### Information sources

Search strategies have been developed using medical subject headings (MeSH), where applicable, and text words related to our condition and outcomes of interest. We will search the following databases: Medline, Embase, CINAHL, Web of Science, Scopus and CENTRAL. Full search strategies for each database can be found in Supplemental Information S1 File. The electronic database search will be supplemented by scanning the reference lists of included and other relevant review studies to capture any additional eligible cited articles not identified in the database searches. Grey literature searches will also be conducted, using OpenGrey, OpenAIRE and OpenDOAR. Where data in studies identified for inclusion are incomplete, study authors will be contacted.

### Search strategy

The specific search strategies (Supplemental Information S1 File) have been developed through an iterative process with input from a Health Sciences Librarian with expertise in systematic review searching. The first search strategy was developed for the MEDLINE database. This has been peer-reviewed by an independent researcher not otherwise associated with the project, using the Peer Review of Electronic Search Strategies (PRESS) standard [20]. The finalized version of this search will be subsequently adapted to fit the syntax and subject headings of the other databases mentioned above. Specific search strategies for each database can be found at the end of this document (Appendix 1). The search will be updated towards the end of the review to ensure any newly published studies are included in the final review.

Additionally, the International Clinical Trials Registry Platform Search Portal and ClinicalTrials.gov will be searched for recently completed trials that might also report on physical associations with CSCR. PROPSERO will also be searched for recently completed systematic reviews on the topic.

### Study records

#### Data management.

Citation abstracts from literature search results will be uploaded to Rayyan (www.rayyan.ai) [21], an AI-powered systematic review management platform. Screening will be performed via this platform, which allows collaboration among reviewers during the study selection process. All databases searched will be uploaded into one pool of abstracts on Rayyan. Duplicates will be detected by the software, manually reviewed by the team and removed.

#### Selection process.

Study titles and abstracts will be screened on the Rayyan platform independently by two reviewers. The team will design screening questions based on eligibility criteria. Screening questions will be reported in the final write up of the systematic review. Screening will be done using these screening questions. Full reports for all titles that appear to meet the eligibility criteria, or where there is disagreement between the first two reviewers, will be obtained. Full text screening of will be conducted using the same screening questions as in the abstract screening stage, independently by the same two reviewers. If further information is required about a study in order to resolve disagreements about eligibility, we will seek this from study authors. Disagreements will be resolved through discussion and, if required, by an independent third reviewer (a senior retinal clinician). None of the reviewers will be masked to journal titles, study authors or institutions.

#### Data collection process.

Data extraction forms will be created on Microsoft Excel. They will be developed prior to screening and will be included in the published protocol. Two reviewers will extract data independently and in duplicate from each eligible study. To ensure consistency between reviewers and ensure familiarity with the data extraction process, a pilot calibration exercise will be conducted on a small selection before starting the full data extraction process. Discrepancies between data extracted by each reviewer will be reviewed by the third reviewer to ensure accuracy. Where data are missing from included studies, the reviewers will contact the study authors with a maximum of three email attempts, to obtain missing information. If multiple reports of a single study are identified during the data extraction process, reviewers will rationalise data extraction into one entry only, and compare individually extracted data points for consistency, with discrepancies resolved by discussion between reviewers and review of all reports of the study in question, and if necessary, by a third reviewer.

### Data Items

Data will be extracted from studies under the following categories:

- Publication details (including study design, number of participants, country, year, setting)
- Patient/participant/population demographics (including age, sex, ethnicity, occupation)
- Past medical history
- Drug history
- Ophthalmic characteristics
- Genetic markers
- Hormonal markers
- Metabolic markers
- Drug/toxin-related markers
- Other reported associations
- Odds or hazards ratios (if reported in studies, with details of any adjustment for confounders)

### Outcomes and prioritisation

Our main outcome of interest is the identification of systemic physical factors associated with CSCR. As such, data on genetic, hormonal, metabolic and drug/toxin-related factors associated with disease will be sought as a priority. The identification of any relationship(s) between risk factor(s) and disease severity is a secondary, lower priority outcome.

### Risk of bias assessment

Risk of bias will be assessed at individual study level, and not at individual risk factor level because this is not practical with the large number of outcomes being collected. The Newcastle-Ottawa Scale [22] will be used for assessing the risk of bias of observational studies. Although risk of bias assessment tools for interventional studies exist, they are focused on the efficacy of the intervention on study outcomes, rather than baseline risk factors for disease as this review is focused on, and so we will also apply the Newcastle-Ottawa Scale [22] to interventional studies, where possible. Studies will be grouped into three categories based on their score on the scale; 0–2 = poor quality, high risk of bias; 3–6 = fair quality, fair risk of bias; 7–9 = high quality, low risk of bias. Two independent reviewers will assess the risk of bias in duplicate for each included study. Disagreements will be discussed between the two reviewers, and if an agreement is not then reached, settled by arbitration from a third independent reviewer. Reviewers will not be masked to studies, as they will have already extracted data from them and so are likely to recognize individual publications. If there is insufficient detail reported in the study to make a judgement, further information will be sought from the study authors and a definitive assessment made with that information. If it is not possible to obtain the required information, then the risk of bias will be labelled as ‘unclear risk’.

### Data synthesis and meta-analysis

The results of this systematic review will be reported according to the PRIMSA statement [23]. Only studies whose control groups are similar to cases except in relation to disease experience, i.e., that confer a low risk of bias, will be included in the meta-analysis. The risk of bias of control groups will be assessed as part of overall risk of bias assessment described above. Any studies in which the control group is identified as being at ‘high’ risk of bias will be excluded from the meta-analysis. Finally, of these studies, only those whose designs are sufficiently homogenous (I^2^ ≤ 50%) will be included in the meta-analysis. Effect sizes will be reported as odds ratios. If different effect measures are used in some studies that are otherwise appropriate for meta-analysis, then appropriate conversions will be used if possible. Odds ratios will be pooled into a random-effects model. The results of meta-analysis will be illustrated using forest plots. Where a meta-analysis is not possible, data will be synthesized by a narrative description of study results, in accordance with Synthesis Without Meta-Analysis (SWiM) guidelines [24].

### Meta-bias

Publication bias across studies included in the meta-analysis will be assessed if sufficient (10+) studies are included. Publication bias will be expressed with funnel plots (study effects expressed as odds ratio vs. sample size), Egger’s linear regression test, and by comparing published reports with proposed outcomes in the protocol of studies where available.

### Confidence in cumulative estimate

The strength of the body of evidence reported in this systematic review will be reported using the Grading of Recommendations Assessment, Development and Evaluation (GRADE) checklist [25].

## Supporting information

S1 File Search Strategies. (DOCX)

S2 File PRISMA P Checklist [26]. (DOCX)
