# Supplementary material for: Physical risk factors associated with central serous chorioretinopathy: A protocol for a systematic review and meta-analysis
Source: PLoS One. 2026 Oct 29;21(10):e0349473. doi: 10.1371/journal.pone.0349473 (PMC13718948; doi:10.1371/journal.pone.0349473)
Supplement: Supplementary file 1 [file pone.0349473.s001.docx]

# Appendix 1: Search strategies

**Embase**

1.	exp central serous retinopathy/

2.	central serous retinopath*.tw.

3.	central serous chorioretinopath*.tw.

4.	central recurrent retinitis.tw.

5.	idiopathic flat detachment of the macula.tw.

6.	angiospastic retinopath*.tw.

7.	capillarospastic central retinitis.tw.

8.	central serous pigment epitheliopath*.tw.

9.	pachychoroid.tw.

10.	or/1-9

11.	risk factor/

12.	risk factor*.tw.

13.	hazard*.tw.

14.	exp gene/

15.	exp genetic predisposition/

16.	exp genome-wide association study/

17.	(gene* or geno*).tw.

18.	exp hormone/

19.	hormon*.tw.

20.	exp metabolism/

21.	metaboli*.tw.

22.	exp physiology/

23.	physiolog*.tw.

24.	exp drug/

25.	exp biological product/

26.	drug*.tw.

27.	medicine*.tw.

28.	medication*.tw.

29.	(biopharmaceutical* or biologic*).tw.

30.	exp pharmacology/

31.	pharmacolog*.tw.

32.	exp toxin/

33.	toxi*.tw.

34.	exp steroid/

35.	exp anabolic agent/

36.	steroid*.tw.

37.	exp epidemiology/

38.	epidemiolo*.tw.

39.	exp causality/ or exp causality assessment/

40.	(aetiolog* or etiolog).tw.

41.	pathog*.tw.

42.	caus*.tw.

43.	mechanis*.tw.

44.	or/11-43

45.	10 and 44

**MEDLINE**

1.	exp Central Serous Chorioretinopathy/

2.	central serous retinopath*.tw.

3.	central serous chorioretinopath*.tw.

4.	central recurrent retinitis.tw.

5.	idiopathic flat detachment of the macula.tw.

6.	angiospastic retinopath*.tw.

7.	capillarospastic central retinitis.tw.

8.	central serous pigment epitheliopath*.tw.

9.	pachychoroid.tw.

10.	or/1-9

11.	Risk Factors/

12.	risk factor*.tw.

13.	hazard*.tw.

14.	exp Genes/

15.	exp Genetic Predisposition to Disease/

16.	exp Genome-Wide Association Study/

17.	(gene* or geno*).tw.

18.	exp Hormones/

19.	hormon*.tw.

20.	exp Metabolism/

21.	metaboli*.tw.

22.	exp Physiology/

23.	physiolog*.tw.

24.	exp Pharmaceutical Preparations/

25.	exp Biological Products/

26.	drug*.tw.

27.	medicin*.tw.

28.	medication*.tw.

29.	(biopharmaceutical* or biologic*).tw.

30.	exp Pharmacology/

31.	pharmacolog*.tw.

32.	exp Toxins, Biological/

33.	toxi*.tw.

34.	exp Steroids/

35.	exp Anabolic Agents/

36.	exp Anabolic Androgenic Steroids/

37.	steroid*.tw.

38.	exp Epidemiology/

39.	epidemiolo*.tw.

40.	exp Causality/

41.	(aetiolog* or etiolog).tw.

42.	pathog*.tw.

43.	caus*.tw.

44.	mechanis*.tw.

45.	or/11-44

46.	10 and 45

**CINAHL**

((MH "Risk Factors+") OR (XB ("risk factor*")) OR (XB hazard*) OR (MH (MH "Genes+" OR MH "Genome Wide Association Study") OR XB ((gene* OR geno*))) OR (MH (MH "Hormones+") OR XB (hormon*)) OR (MH (MH "Metabolism+") OR XB (metaboli*)) OR (MH "Physiology+" OR physiolog*) OR (MH (MH "Drugs+") OR XB (drug* OR medicin* OR medication*)) OR (MH (MH "Biological Products+") OR XB (biopharmaceutical* OR biologic*)) OR ((MH "Pharmacological and Biological Treatments+") OR pharmacolog*) OR (MH (MH "Toxins+") OR XB (toxi*)) OR (MH (MH "Steroids+" OR MH "Anabolic Steroids") OR XB (steroid*)) OR ((MH "Epidemiology+") OR XB (epidemiolo*)) OR (MH (MH "Causal Attribution" OR MH "Causality") OR XB (caus* OR aetiolog* OR etiolog* OR pathog* OR mechanis*))) AND (XB ("central serous chorioretinopath*" OR "central serous retinopath*" OR "central recurrent retinitis" OR "idiopathic flat detachment of the macula" OR "angiospastic retinopath*" OR "capillarospastic central retinitis" OR "central serous pigment epitheliopath*" OR pachychoroid))

**Web of Science**

**ALL=(“central serous chorioretinopath*” OR “central serous retinopath*” OR “idiopathic flat detachment of the macula” OR “angiospastic retinopath*” OR “capillarospastic central retinitis” OR “central serous pigment epitheliopath*” OR pachychoroid) AND ALL=(“risk factor*” OR hazard* OR gene* OR geno* OR “genetic predisposition” OR “genome-wide association study” OR hormon* OR metaboli* OR physiolog* OR drug* OR “biological product” OR medicin* OR medication* OR biopharmaceutical* OR biologic* OR pharmacolog* OR toxi* OR steroid* OR “anabolic agent” OR epidemolo* OR causality OR aetiolog* OR etiolog* OR pathog* OR caus* OR mechanis*)**

**Scopus**

( TITLE-ABS-KEY ( "central serous chorioretinopath*" OR "central serous retinopath*" OR "idiopathic flat detachment of the macula" OR "angiospastic retinopath*" OR "capillarospastic central retinitis" OR "central serous pigment epitheliopath*" OR pachychoroid ) ) AND ( TITLE-ABS-KEY ( "risk factor*" OR hazard* OR gene* OR geno* OR "genetic predisposition" OR "genome-wide association study" OR hormon* OR metaboli* OR physiolog* OR drug* OR "biological product" OR medicin* OR medication* OR biopharmaceutical* OR biologic* OR pharmacolog* OR toxi* OR steroid OR "anabolic agent" OR epidemolo* OR causality OR "causality assessment" OR aetiolog* OR etiolog* OR pathog* OR caus* OR mechanis* ) )

**Cochrane / CENTRAL**

(central serous chorioretinopathy) OR (central NEXT serous NEXT chorioretinopath*) OR (central NEXT serous NEXT retinopath*) OR (idiopathic flat detachment of the macula) OR (angiospastic NEXT retinopath*) OR (capillarospastic central retinitis) OR (central NEXT serous NEXT pigment NEXT epitheliopath*) OR pachychoroid
